# Light-Activated Elongation/Shortening and Twisting of a Nematic Elastomer Balloon

**DOI:** 10.3390/polym14061249

**Published:** 2022-03-20

**Authors:** Lin Zhou, Yujie Wang, Kai Li

**Affiliations:** 1 School of Mechanical and Electrical Engineering, Anhui Jianzhu University, Hefei 230601, China; zhoulin@ahjzu.edu.cn; 2 Department of Civil Engineering, Anhui Jianzhu University, Hefei 230601, China; yjwang_@outlook.com

**Keywords:** nematic elastomer, balloon, optically-responsive, contraction, twisting

## Abstract

Nematic elastomer balloons with inflation-induced axial contraction and shear/torsion effect can be used as actuators for soft robots, artificial muscles, and biomedical instruments. The nematic elastomer can also generate drastic shape changes under illumination, and thus light can be utilized to activate the deformation of nematic elastomer balloons with huge advantages of being accurate, fast, untethered, and environmentally sustainable without chemical byproducts. To explore light-activated deformation behaviors of the balloon, a phenomenological relationship between light intensity and material parameters describing polymer backbone anisotropy is proposed from experiments, and a theoretical model of an optically-responsive nematic elastomer balloon is established based on the nematic elastomer theory. Various light-activated elongation/shortening and twisting behaviors in the cases of free-standing and axial-loading are presented and their mechanisms are elucidated. The light intensity and initial mesogen angle have great influences on the light-activated deformations including the radius, length, shearing angle and mesogen angle. Light can be easily controlled to trigger rich deformation processes, including elongation/shortening and torsion. The results of this paper are expected to promote the understanding of the light-activated deformation behaviors of the nematic elastomer balloon, and the applications in light-activated actuators and machines.

## 1. Introduction

Nematic elastomer represents a new type of polymer composite intelligent material, which is moderately crosslinked with non-crosslinked liquid crystal polymer and exhibits elasticity during the transition from isotropic state to liquid crystal state [1]. The mechanical behaviors are determined by the stretch of polymer chains as well as rotation of liquid crystal mesogens within it [2]. The self-organization of liquid crystal systems and the flexibility brought by elasticity of the polymer network allow large and reversible anisotropic dimensional changes under external stimulations. The nematic elastomer bears the most abundant mechanical properties and has been studied extensively [3,4,5,6,7]. Recently, nematic elastomer has been widely used in the fields of micro/nano devices, sensors and actuators [8,9,10,11,12,13,14].

Nematic elastomer tubes or balloons are often used to build artificial muscles which can be utilized in robotic arms, prosthetics, medical robots [15,16,17,18]. Many intriguing phenomena associated with large deformation of balloons have been observed and intensively studied in the past several decades [19,20,21,22]. He et al. [15] experimentally analyzed the anomalous inflation phenomenon of the cylindrical nematic elastomer balloons when subjected to an additional axial load and adopted a theory based on the quasiconvex free energy to understand the inflation behaviors. Theoretical research on the inflation-induced torsion of a cylindrical nematic elastomer balloon under the combined action of axial loading and inflating pressure was carried out by Li et al. [23]. The inflation process of the nematic elastomer balloon shows various performances in the cases of different axial tensions. Similar to the routine balloon, a bifurcation condition is valid by Maxwell’s equal-area rule and the localized bulge can take place in the inflating process [24,25,26]. It was found that the existence of disconnected solution branches and the bifurcation may undergo a jump from one branch to another, which is accompanied with the director reorientation similar to the uniaxial nematic elastomers [27] or bent nematic liquid crystal elastomers [28,29].

Through integrating photoisomerizable molecules into nematic elastomer, large and reversible deformations of the nematic elastomer composite material can be induced optically [1,30,31,32,33,34,35,36,37]. The sufficient illumination can change the nematic order through isotropic–nematic phase transition and produce reorientation of the liquid crystal mesogens. The isotropic-to-nematic phase transition provides nematic elastomer with an unusual soft elasticity and generates shape changes under light illumination [38,39,40,41,42]. Recently, experiments were conducted on a range of different photoactive nematic elastomers and focused on describing the specific actuation modes such as periodic behavior and bending/uniaxial contraction effects of nematic films [43,44,45,46]. In order to explain its behaviors and development for photo-induced deformation, many kinds of theoretical models have been proposed [1,43,47]. Bai et al. [48] explored the mechanical behavior of a photoactive nematic elastomer under both light intensity and external force. He et al. [49] conducted the theory of uniaxial tension of a nematic elastomer film with different initial mesogen angles. Bladon et al. [50] demonstrated an entirely new nematic phase transition and showed that the shear was related to the internal nematic direction during its rotation. Due to the elastic interaction between the liquid crystal mesogens, the initial mesogen angle plays an important role in the shearing behavior of the nematic elastomer film under uniaxial tension.

Despite the intensive studies of the inflation-induced deformation of the nematic elastomer balloon, there has been far less research on the light-driven deformation behaviors. The nematic elastomer balloon may present axial contraction/expansion and twisting behaviors under the actuation of light, and has potential applications for accurate active control in the fields of soft machines and equipment, due to the huge advantages of being accurate, fast, untethered, and environmentally sustainable without chemical byproducts. Based on the experimental results of light-activated deformation of nematic elastomers, this paper proposes a phenomenological relationship between the light intensity and the light-dependent material parameter describing the polymer backbone anisotropy. Then, we establish a new theoretical model of optically-responsive nematic elastomer combined with the free energy proposed by Bladon et al. [50] and theoretically study the light-activated deformation of a nematic balloon with axial load.

This paper is written as follows. Firstly, we formulate the governing equations for the deformation of the cylindrical nematic elastomer balloon in Section 2. Secondly, the light-activated elongation/shortening and twisting behaviors under free-standing case are revealed and their mechanisms are elucidated in Section 3. Thirdly, we focus on three kinds of light-activated deformation behaviors under axial-loading case in Section 4. Finally, the conclusion of this paper is summarized.

## 2. Modelling of an Optically-Responsive Nematic Elastomer Balloon

**Figure 1:**
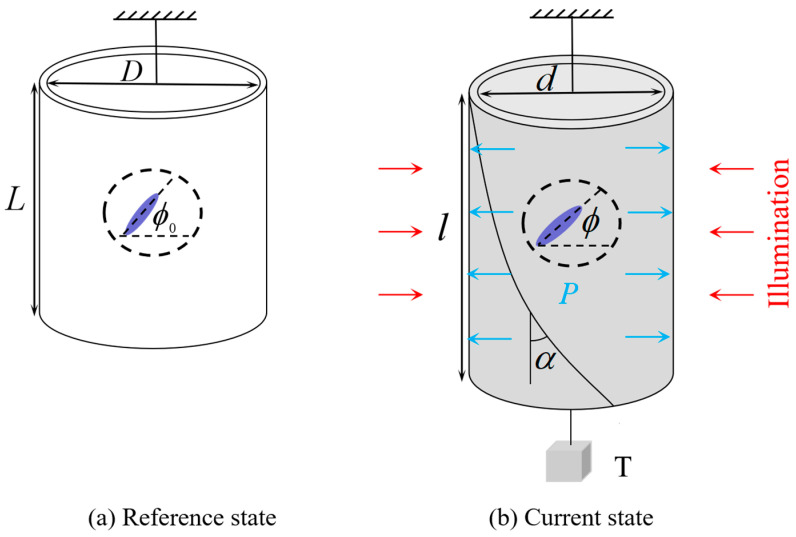
A cylindrical nematic elastomer balloon subjected to external axial load T and internal inflating pressure P under illumination with light intensity.

Figure 1 sketches a cylindrical nematic elastomer balloon subjected to external axial load T and internal inflating pressure P under illumination with light intensity. The cylindrical balloon is made of nematic elastomer combing mesogens and polymer network in molecular scale, which is synthesized through a two-step crosslinking polymerization method [15,49]. Through incorporating photoactive molecules into nematic liquid crystal elastomers, photoactive nematic elastomers can deform when illuminated. To formulate the governing equations of the light-activated deformation and reveal its mechanism, we set the stress-free state as the reference state, in which the length of the nematic elastomer balloon is L, the cross-section diameter is D, the thickness is H, the volume is $V_{0}$, and the initial mesogen angle is $\phi_{0}$. In the current state, the nematic balloon is subjected to inflating pressure P, axial load T and light intensity I. The length becomes l, the cross-section diameter becomes d, the thickness becomes h, the volume changes to v, and the mesogen angle is ϕ. Due to the rotation of the liquid crystal mesogens, the upper and lower end faces of the balloon rotate relative to each other and the balloon shears with shearing angle α. For simplicity, we assume that the balloon length is much larger than the cross-section diameter, so the end effect can be ignored. In addition, the cross-section diameter is assumed to be larger than its thickness, and membrane assumption is applied. Therefore, the nematic elastomer balloon is in a homogeneous state.

### 2.1. Governing Equations for the Light-Activated Deformation Behaviors of a Nematic Elastomer Balloon

To understand the light-activated deformation behaviors of the nematic elastomer balloon, we can derive the governing equations according to the well-known nematic elastomer theory proposed by Bladon et al. [50]. The function of the free energy density can be given as

(1) $$W\left(F,\theta \right)=\frac{\mu }{2}\mathrm{tr}\left(l_{0}F^{T}l^{-1}F\right)$$

where μ is the shear modulus and F is the deformation gradient tensor in the nematic balloon, which can be written as [51,52,53]

(2) $$F=\left[\begin{array}{ccc} \lambda_{z} & 0 & 0\\ \lambda_{z}\tan\alpha & \lambda_{\theta } & \\ 0 & 0 & \lambda_{r} \end{array}\right]$$

where $\lambda_{z}$,$\lambda_{\theta }$ and $\lambda_{r}$ are extension ratios as follows:

(3) $$\lambda_{z}=l/L,\;\lambda_{\theta }=d/D,\;\lambda_{r}=h/H$$

We assume that the nematic elastomer is incompressible during the deformation of the balloon, namely,

(4) $$\lambda_{\theta }\lambda_{z}\lambda_{r}=1$$

$l_{0}$ and l are the polymer chain configurations of the nematic elastomer before and after deformation, which can be represented by

(5) $$l_{0}=\delta +\left(r-1\right)n_{0}n_{0}$$

And

(6) l=δ+(r−1)nn

where δ is the unit tensor, r is the specific material parameter which will be given in detail in Section 2.2, and its value depends on the light intensity. $n_{0}$ and n are the initial and current mesogen directors before and after deformation, which can be written as

(7) $$n_{0}=\left(\sin\phi_{0},-\cos\phi_{0},0\right)$$

And

(8) n=(sinϕ,−cosϕ,0)

where $\phi_{0}$ and ϕ are the initial and current mesogen angles between the mesogens and the axis of the balloon. Hence, it is found that the free energy of the nematic elastomer is affected by the above four independent parameters as $W=W\left(\lambda_{\theta },\lambda_{z},\alpha ,\phi \right)$. In addition, during the deformation of the nematic balloon, the equilibrium equation can be given according to the second law of thermodynamics at a constant temperature as follows:

(9) $$\delta W-s_{iK}\delta F_{iK}=0$$

where $s_{iK}$ is the nominal stress applied on the elastomer, δW and $\delta F_{iK}$ are infinitesimal variations of free energy of the elastomer and the deformation gradient. Considering that the non-zero components of nominal stress are $s_{\theta \theta }$ and $s_{zz}$, the Equation (9) can be written as

(10) $$\frac{\partial W}{\partial \lambda_{\theta }}\delta \lambda_{\theta }+\frac{\partial W}{\partial \lambda_{z}}\delta \lambda_{z}+\frac{\partial W}{\partial \alpha }\delta \alpha +\frac{\partial W}{\partial \varphi }\delta \phi -s_{\theta \theta }\delta \lambda_{\theta }-s_{zz}\delta \lambda_{z}=0$$

The equilibrium equation can be further obtained that

(11) $$\frac{\partial W\left(\lambda_{\theta },\lambda_{z},\phi ,\alpha \right)}{\partial \lambda_{\theta }}=s_{\theta \theta }$$

(12) $$\frac{\partial W\left(\lambda_{\theta },\lambda_{z},\phi ,\alpha \right)}{\partial \lambda_{z}}=s_{zz}$$

(13) $$\frac{\partial W\left(\lambda_{\theta },\lambda_{z},\phi ,\alpha \right)}{\partial \phi }=0$$

(14) $$\frac{\partial W\left(\lambda_{\theta },\lambda_{z},\phi ,\alpha \right)}{\partial \alpha }=0$$

The radial stress can be ignored due to the thickness of the balloon being much smaller than the cross-section diameter. The balance of axial and hoop stresses can be expressed as

(15) $$\sigma_{\theta \theta }=\frac{Pd}{2h},\sigma_{zz}=\frac{Pd}{4h}+\frac{T}{\pi dh}$$

(16) $$s_{\theta \theta }=\frac{PD}{2H}\lambda_{\theta }\lambda_{z},s_{zz}=\frac{PD}{4H}\lambda_{\theta }^{2}+\frac{T}{\pi DH}$$

To nondimensionalize the governing equations above, we introduce the following dimensionless quantities $\bar{P}=\frac{PD}{\mu H}$ and $\bar{T}=\frac{2T}{\pi \mu DH}$. A combination of Equations (1), (11)–(14) and (16) gives the governing equations for the light-activated deformation behaviors of the balloon:

(17) $$\begin{array}{l} \bar{P}\lambda_{\theta }\lambda_{z}=\frac{2\left(r\cos^{2}\phi_{0}+\sin^{2}\phi_{0}\right)\left(r\sin^{2}\phi +\cos^{2}\phi \right)}{r}\lambda_{\theta }\\ -\frac{{\left(r-1\right)}^{2}\sin\left(2\phi \right)+2\left(r-1\right)\tan\alpha \left(r\sin^{2}\phi +\cos^{2}\phi \right)}{2r}\sin\left(2\phi_{0}\right)\lambda_{z}-\frac{2}{\lambda_{z}^{2}\lambda_{\theta }^{3}} \end{array}$$

(18) $$\begin{array}{l} \frac{\bar{P}}{2}\lambda_{\theta }^{2}+\bar{T}=\frac{\left(r\sin^{2}\phi_{0}+\cos^{2}\phi_{0}\right)\left[\left(r-1\right)\cos\left(2\alpha -2\phi \right)+r+1\right]}{r\cos^{2}\alpha }\lambda_{z}\\ -\frac{\left(r-1\right)\sin\left(2\phi_{0}\right)\left[\left(r+1\right)\sin\alpha +\left(r-1\right)\sin\left(2\phi -\alpha \right)\right]}{2r\cos\alpha }\lambda_{\theta }-\frac{2}{\lambda_{z}^{3}\lambda_{\theta }^{2}} \end{array}$$

(19) $$\frac{\left(r\sin^{2}\phi_{0}+\cos^{2}\phi_{0}\right)\sin\left(2\alpha -2\phi \right)}{\cos^{2}\alpha \left(r\cos^{2}\phi_{0}+\sin^{2}\phi_{0}\right)}\lambda_{z}^{2}-\frac{\left(r-1\right)\sin\left(2\phi_{0}\right)\cos\left(2\phi -\alpha \right)}{\cos\alpha \left(r\cos^{2}\phi_{0}+\sin^{2}\phi_{0}\right)}\lambda_{z}\lambda_{\theta }+\sin\left(2\phi \right)\lambda_{\theta }^{2}=0$$

(20) $$\frac{\left(r\sin^{2}\phi_{0}+\cos^{2}\phi_{0}\right)\left[\left(r-1\right)\sin\left(2\phi -\alpha \right)+\left(r+1\right)\sin\alpha \right]}{\cos\alpha \left(r\sin^{2}\phi +\cos^{2}\phi \right)}\lambda_{z}-\left(r-1\right)\sin\left(2\phi_{0}\right)\lambda_{\theta }=0$$

The above formula can predict the light-activated deformation of the nematic elastomer balloon, but the relationship between the material parameter r and the light intensity is required, which will be further discussed as follows.

### 2.2. Light-Dependent Material Parameter r

**Figure 2:**
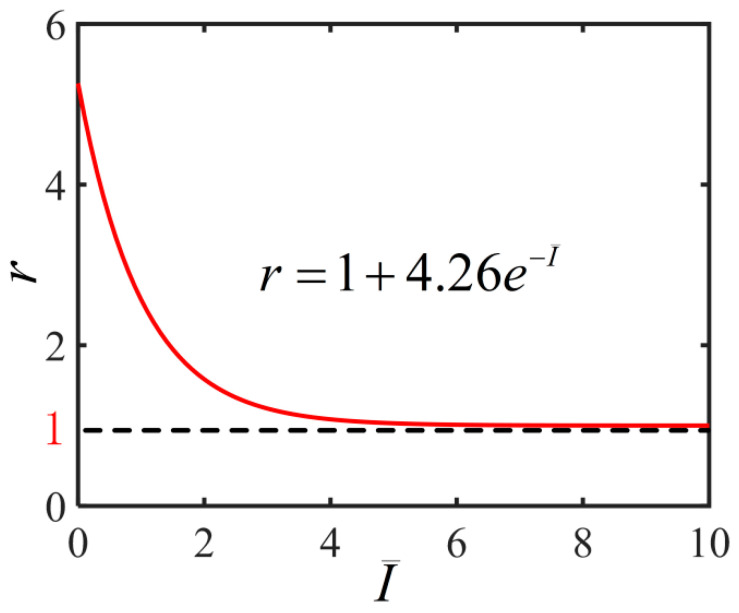
Variation of the light-dependent material parameter r with the light intensity $\bar{I}$ for the cylindrical nematic elastomer balloon subjected to illumination.

Experiments show that the deformation of the nematic elastomer depends on the light intensity [34,35,36,37]. To explore the light-activated deformation of the nematic elastomer balloon, we need to establish the relationship between light intensity I and the material parameter describing the polymer backbone anisotropy r. For example, the typical value of parameter r is $r_{L}=5.26$ without light illumination in the experiments [15,49]. When the light is infinite and the polymer backbone becomes isotropic, i.e., r=1. Based on the experiments, we propose the following phenomenological relationship:

(21) $$r=1+\left(r_{L}-1\right)e^{-\bar{I}}$$

where $\bar{I}=I/I_{0}$ with $I_{0}$ being characteristic light intensity. As shown in the Figure 2, with the increase of light intensity, the material parameter r gradually decreases from $r_{L}$ to 1. This means that the degree of anisotropy of the polymer backbone gradually decreases to isotropy, thus causing deformation of the balloon.

### 2.3. Solution Method

To understand the light-activated deformation behaviors of the nematic elastomer balloon, we can derive the solution of the governing Equations (17)–(21) numerically using MATLAB software. The typical values of material properties and geometric parameters from accessible experiments [15,23,48,49] are listed in Table 1, and the dimensionless parameters are estimated as $\bar{I}=0\sim 6$ and $\bar{T}=0\sim 1$. Hereon, we firstly provide the quantities such as light intensity $\bar{I}$, axial load $\bar{T}$, initial mesogen angle $\phi_{0}$, hoop stretch $\lambda_{\theta }$, and then we can solve the value of the axial stretch $\lambda_{z}$, pressure $\bar{P}$, shearing angle α, and mesogen angle ϕ in the deformed nematic balloon. Hence, the relationships between the variation of each quantity with the volume for different light intensities $\bar{I}$ in the inflation process are further drawn. Furthermore, the relationships between different physical quantities and light intensity are found for a given quantity of inflating gas. We assume that the thickness of the balloon is much thinner than the decay length of light in the nematic elastomer, such that the light intensity is homogeneous. Assuming that the gas inside the balloon is the ideal gas, its equation of state is PV=nRT, where $V=\frac{\pi D^{2}}{4}L\lambda_{\theta }^{2}\lambda_{z}$ is gas volume, *n* is quantity of inflating gas, R is ideal gas constant, and T is the thermodynamic temperature of the ideal gas. By introducing $\bar{P}=\frac{PD}{\mu H}$, $\bar{V}=\lambda_{\theta }^{2}\lambda_{z}$ and $\bar{n}=\frac{4nRT}{u\pi HD}$, the dimensionless form is $\bar{P}\bar{V}=\bar{n}$.

**Figure 3:**
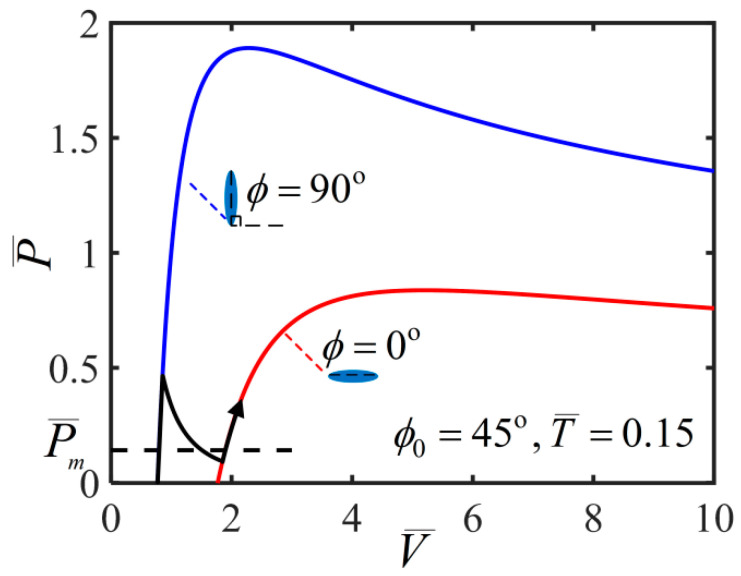
Pressure vs. volume during the inflation process for $\bar{T}=0.15$ and $\phi_{0}=45^{\circ }$, in which the blue curve shows the solution of $\phi =90^{\circ }$ and the red curve shows the solution of $\phi =0^{\circ }$. The black N-shaped curve shows a typical inflation process by volume control.

In the process of solving nonlinear governing equations, we find that there are multiple solutions as shown in the Figure 3, which plots the variation of inflating pressure with the volume for $\bar{T}=0.15$ and $\phi_{0}=45^{\circ }$. Here, we briefly introduce the solution method as previously described by Li et al. [23]. The stable solution is according to the smaller potential energy of the multiple equilibrium states [23]. The blue curve in Figure 3 shows the solution of $\phi =90^{\circ }$ and the red curve shows the solution of $\phi =0^{\circ }$. In addition, the inflating pressure exhibits up/down/up/down behavior with the volume which represents a typical inflation process as shown by the black curve. The previous research shows that a bifurcation condition is valid by Maxwell’s equal-area rule and the localized bulging in an inflated cylindrical balloon can take place [23,24,25,26]. There is an inhomogeneous and complex state during the localized bulging in the balloon. In the following discussion, we skip the details of the localized bulging and bulge propagation to investigate the light-activated deformation behaviors based on homogeneous analysis by assuming that the film is uniformly deformed.

## 3. Light-Activated Contraction and Twisting of a Free-Standing Nematic Elastomer Balloon

**Figure 4:**
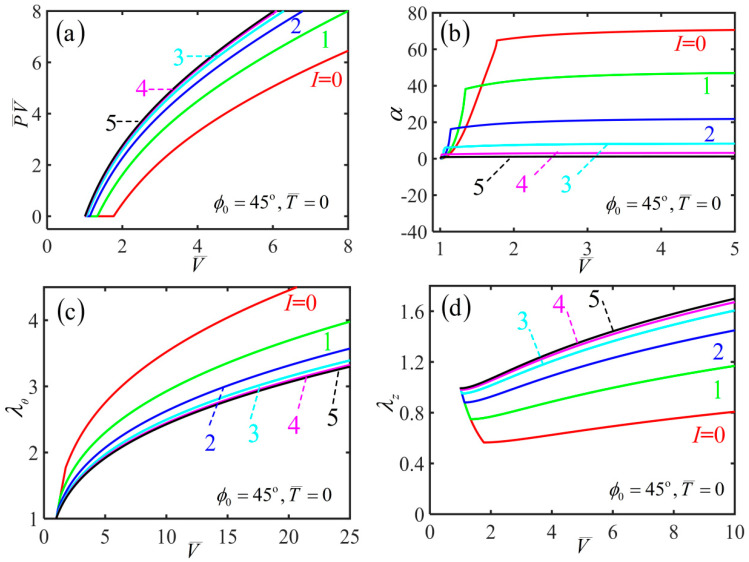
Variation of (**a**) $\bar{P}\bar{V}$, (**b**) shearing angle, (**c**) hoop stretch and (**d**) axial stretch with the volume during the inflation process of a free-standing nematic elastomer balloon for different light intensities.

To investigate light-activated deformation behaviors of the cylindrical free-standing nematic elastomer balloon under illumination, we first plot the variations of various physical quantities including $\bar{P}\bar{V}$ (i.e., the quantity $\bar{n}$ of inflating gas), hoop stretch $\lambda_{\theta }$, axial stretch $\lambda_{z}$ and shearing angle α with the volume in the inflation process for initial mesogen angle $\phi_{0}=45^{\circ }$ under different light intensities $\bar{I}$, as shown in Figure 4. Figure 4a shows that with the increase of the volume, the quantity $\bar{P}\bar{V}$ keeps at zero. As the volume increases further, $\bar{P}\bar{V}$ increases sharply when the volume exceeds a critical value ${\bar{V}}_{\mathrm{crit}}$, and this typical inflating process is similar to the reported experiments [15]. Meanwhile, ${\bar{V}}_{\mathrm{crit}}$ decreases with the increase of the light intensity. Figure 4b shows that with the increase of the volume, the shearing angle increases monotonously, and gradually approaches an asymptotic angle $\alpha_{\mathrm{asym}}$. With the increase of light intensity, $\alpha_{\mathrm{asym}}$ decreases asymptotically to zero.

**Figure 5:**
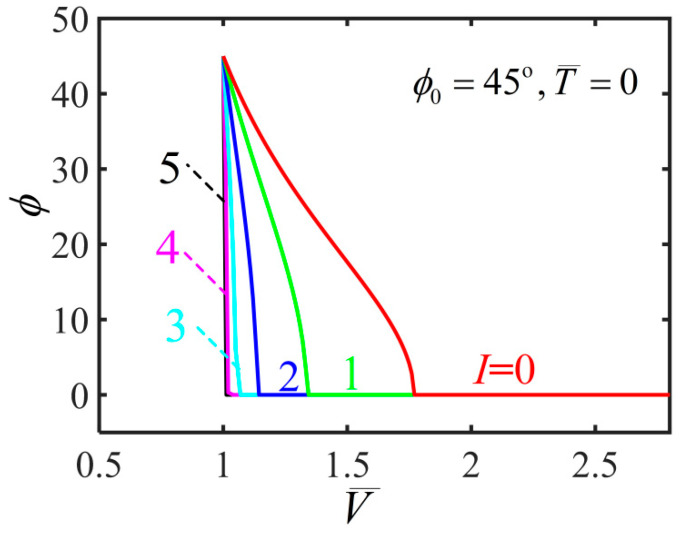
The variation of mesogen angle with the volume during the inflation process of a free-standing nematic elastomer balloon for different light intensities.

Figure 4c,d show that for a given light intensity, the radius of balloon initially increases significantly, while the length of the balloon decreases significantly. This inflation-induced axial contraction is consistent with the reported experiments [15], which is far different from the case of routine balloons [19,20,21,22]. During the further inflation process, both the radius and length of the balloon increase, and the mesogen angle becomes locked at $0^{\circ }$, as shown in Figure 5 plotting the variation of mesogen angle with the volume during the inflation process. With the increase of light intensity, the radius decreases while the length increases.

**Figure 6:**
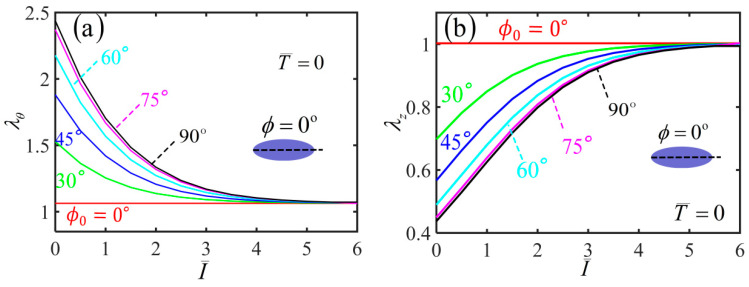
Variations of (**a**) hoop stretch and (**b**) axial stretch with the light intensity of the free-standing nematic elastomer balloon for different initial mesogen angles.

Based on the results of various physical quantities during the inflation process, we further analyze the light-activated deformation behaviors of the free-standing nematic elastomer balloon for the different initial angles under a constant quantity of inflating gas. Figure 6a,b plot the variations of hoop stretch $\lambda_{\theta }$ and axial stretch $\lambda_{z}$ with the light intensity for different initial mesogen angles. For $\phi_{0}=0^{\circ }$, both the radius and length of the balloon remain unchanged with the increase of the light intensity. For nonzero initial mesogen angle, the radius of the non-illuminated balloon has an initial value due to the inflation, which increases with the increase of the initial mesogen angle $\phi_{0}$. For a given nonzero initial mesogen angle, the radius gradually decreases to 1 with the increase of light intensity (Figure 6a). The length of non-illuminated balloon also has an initial value due to the inflation, which decreases with the increase of $\phi_{0}$. For a given initial mesogen angle, the length increases gradually to 1 with the increase of light intensity (Figure 6b).

**Figure 7:**
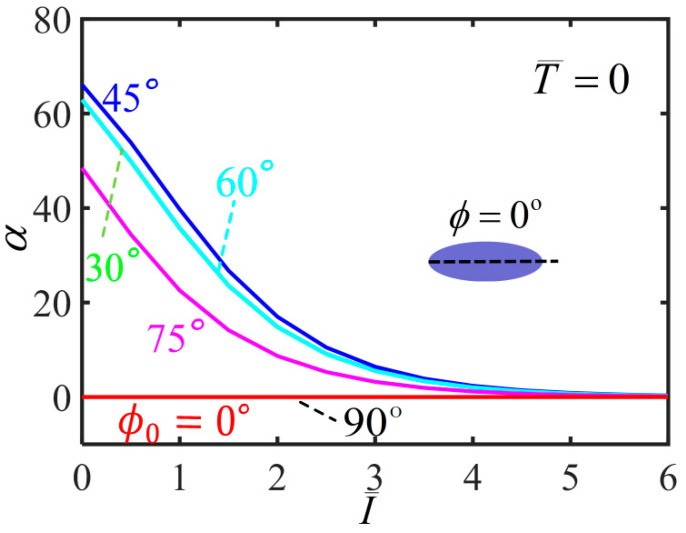
Variations of shearing angle α with the light intensity of the free-standing nematic elastomer balloon for different initial mesogen angles.

Figure 7 plots the variation of shearing angle with the light intensity for different initial mesogen angles. For $\phi_{0}=0^{\circ }$ and $\phi_{0}=90^{\circ }$, the shearing angle of the balloon is always kept at $0^{\circ }$ with the increase of the light intensity. For $0<\phi_{0}<90^{\circ }$, the shearing angle monotonously decreases with the increase of light intensity. For a given light intensity, the shearing angle firstly increases and then decreases with the increase of the initial mesogen angle, which has the maximum at $\phi_{0}=45^{\circ }$. Meanwhile, for a light intensity large enough, the balloon does not shear in the membrane, and the upper and lower end cross-sections do not twist. This is because the material parameter r is close to 1 and the polymer backbone becomes isotropic.

**Figure 8:**
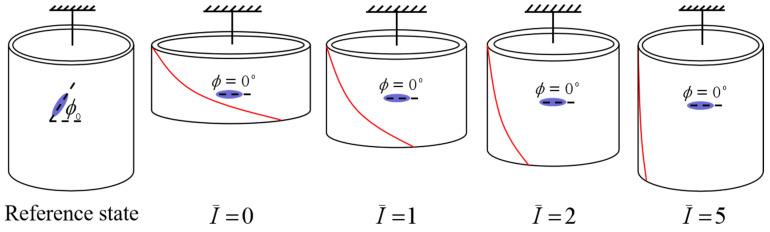
The representative process of a free-standing nematic elastomer balloon for initial mesogen angle $\phi_{0}=45^{\circ }$ under different light intensities.

In order to understand the light-activated deformation process of the balloon, Figure 8 illustrates a typical process of a free-standing nematic elastomer balloon for an initial mesogen angle $\phi_{0}=45^{\circ }$ under different light intensities. From the reference state to the initial non-illuminated state, the radius of balloon increases significantly while the length decreases significantly, which is consistent with the reported experiments [15]. Meanwhile, the mesogen angle ϕ becomes locked at $0^{\circ }$. With the increase of light intensity, the radius decreases close to 1, while the length increases close to 1. The mesogen angle keeps at $0^{\circ }$ and is not affected by illumination. The shearing angle α monotonously decreases with the increase of light intensity.

## 4. Light-Activated Contraction and Twisting of a Nematic Elastomer Balloon Subjected to External Axial Load

**Figure 9:**
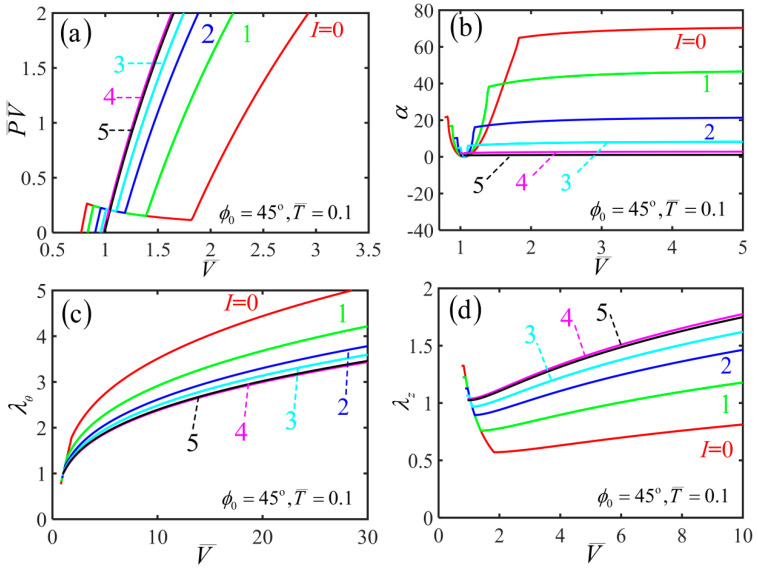
Variations of (**a**) $\bar{P}\bar{V}$, (**b**) shearing angle, (**c**) hoop stretch and (**d**) axial stretch with the volume of the nematic elastomer balloon subjected to axial load $\bar{T}=0.1$ and mesogen angle $\phi_{0}=45^{\circ }$ for different light intensities.

He et al. [15] experimentally studied the inflation behavior of the nematic balloon when it is subjected to an additional axial load. It was found that the axial load represented an important factor associated with the inflation of elastomer balloons. To study the light-activated deformation behaviors of cylindrical nematic balloon with an additional axial load under illumination, we first plot the variations of various physical quantities with the volume in the inflation process for initial mesogen angle $\phi_{0}=45^{\circ }$ and axial load $\bar{T}=0.1$ under different light intensities $\bar{I}$, as shown in Figure 9. Figure 9a shows that the curve is N-shaped, indicating the balloon initially shrinks conspicuously in volume with the additional axial load, and this typical inflating process is similar to the reported experiments [15]. It is an anomalous phenomenon, which is far different from the free-standing state of nematic elastomer balloon, and can be explained with the soft elasticity or zero energy mode deformation [50]. Meanwhile, there is an inhomogeneous and complex state during the localized bulge formation and propagation in the balloon [23,24,25,26]. After the whole balloon is bulged, the $\bar{P}\bar{V}$ increases with the increasing volume, and the mesogen angle is kept at $0^{\circ }$.

Figure 9b shows that, for a given light intensity, the shear angle of the balloon initially decreases, then increases gradually to an asymptotic angle with the inflating air amount increasing. With the increase of inflating air volume, the shearing angle finally gradually approaches an asymptotic value, which decreases with the increase of light intensity. Figure 9c shows that for a given light intensity, there is an obvious initial shortening in the hoop direction of the balloon, and then the hoop stretch increases with the subsequent inflation process. Figure 9d shows that for a given light intensity, the balloon exhibits conspicuous initial elongation in the axial direction. During the subsequent inflation process, the length firstly decreases and then increases with the increase of volume. The results are consistent with the reported experiments [15].

Based on the results of various physical quantities in the inflation process and the N-shaped curve shown in Figure 9a, it is found that the balloon bears three kinds of light-activated deformation behaviors for different initial mesogen angles, which depend on the constant quantity of inflating gas.

(1) The case of small PV

**Figure 10:**
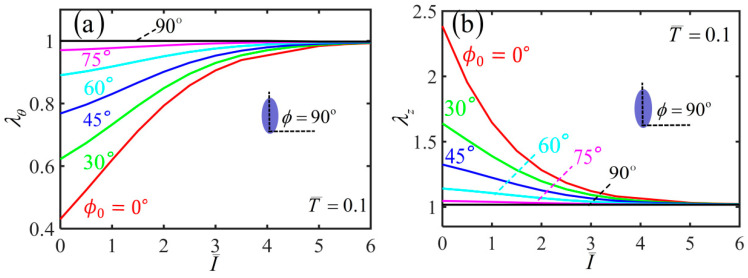
Variation of (**a**) hoop stretch and (**b**) axial stretch with the light intensity of the nematic elastomer balloon with axial load $\bar{T}=0.1$ for different initial mesogen angles.

For a small quantity of inflating gas, Figure 10 plots the variations of hoop stretch $\lambda_{\theta }$ and axial stretch $\lambda_{z}$ with the light intensity for different initial mesogen angles. For $\phi_{0}=0^{\circ }$, both the radius and length of the balloon remain unchanged with the increase of light intensity. For a nonzero initial mesogen angle, the radius of the non-illuminated balloon has an initial value due to the inflation, which increases with the increasing initial mesogen angle. For a given nonzero initial mesogen angle, the radius gradually increases to 1 with the increase of light intensity (Figure 10a). Moreover, the length of non-illuminated balloon also has an initial value due to the inflation, which decreases with the increase of $\phi_{0}$. For a given initial mesogen angle, the length decreases gradually to 1 with the increase of light intensity (Figure 10b).

**Figure 11:**
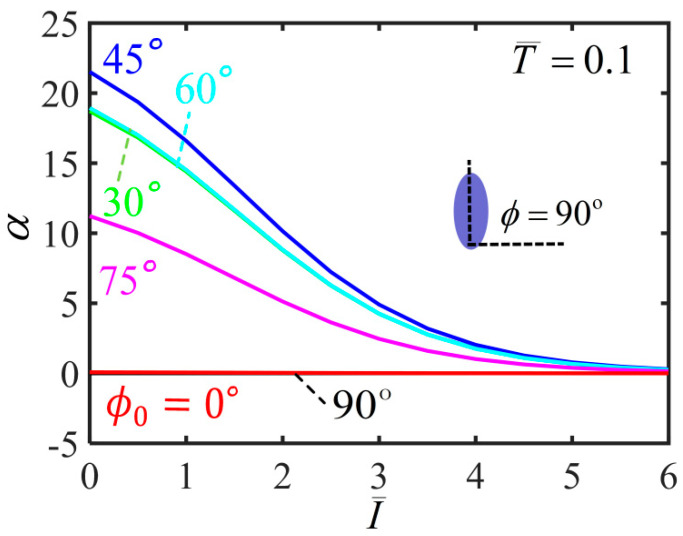
Variation of the shearing angle with the light intensity of the nematic elastomer balloon with axial load $\bar{T}=0.1$ for different initial mesogen angles.

Figure 11 plots the variation of the shearing angle α with the light intensity for different initial mesogen angles. For a given light intensity, the shearing angle firstly increases and then decreases with the increase of the initial mesogen angle, which has the maximum at $\phi_{0}=45^{\circ }$. For $\phi_{0}=0^{\circ }$ and $\phi_{0}=90^{\circ }$, the shearing angle of the balloon is always kept at $0^{\circ }$ with the increase of the light intensity. For $0<\phi_{0}<90^{\circ }$, the shearing angle monotonously decreases to 0 with the increase of light intensity. For infinite light intensity, the balloon does not twist because the polymer backbone becomes isotropic.

**Figure 12:**
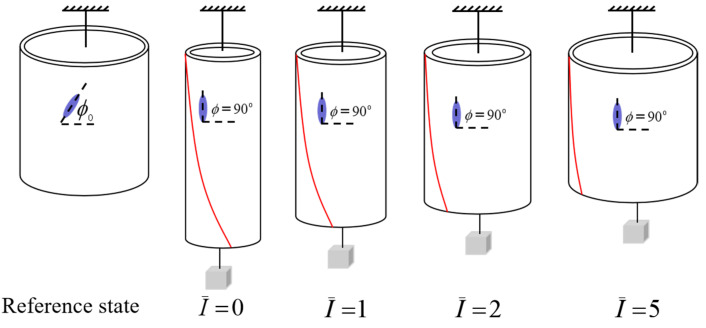
The representative process of a nematic elastomer balloon for initial mesogen angle $\phi_{0}=45^{\circ }$ and axial load $\bar{T}=0.1$ under different light intensities.

Figure 12 illustrates a typical process of a nematic elastomer balloon for initial mesogen angle $\phi_{0}=45^{\circ }$ and axial load $\bar{T}=0.1$. From the reference state to the initial non-illuminated state, the radius of the balloon initially decreases significantly while the length of balloon increases significantly, and the mesogen angle becomes locked at $90^{\circ }$, which is consistent with the reported experiments [15]. With the increase of light intensity, the radius increases and the length decrease asymptotically to 1. The mesogen angle keeps at $90^{\circ }$ and is not affected by the light illumination. The shearing angle monotonously decreases with the increase of light intensity.

(2) The case of medium PV

For a medium quantity of inflating gas, the light-activated deformation of the balloon may accompany the complex process of localized bulging and bulge propagation. The localized bulging balloon is in an inhomogeneous two-phase coexisting state, which is beyond the research scope of this paper. Based on our homogeneous state-based model, we focus on the phase transition between homogeneous and inhomogeneous states in the process of light-activated deformation.

**Figure 13:**
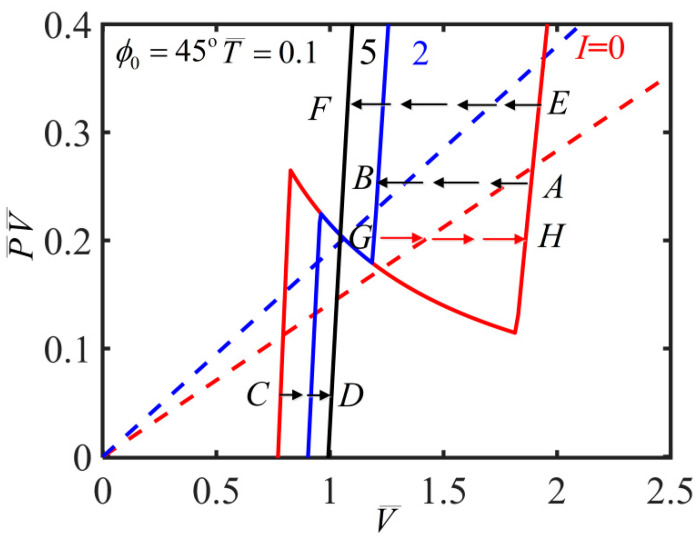
Variations of $\bar{P}\bar{V}$ versus volume of the balloon under different light intensities with initial mesogen angle $\phi_{0}=45^{\circ }$ and axial load $\bar{T}=0.1$.

Figure 13 dedicates the relationship of the value of $\bar{P}\bar{V}$ (i.e., the quantity of inflating gas) and the volume in the inflating process of a nematic elastomer balloon for $\bar{T}=0.1$ and $\phi_{0}=45^{\circ }$ under different light intensities. The dotted lines represent Maxwell’s condition for the coexistence of two phases [23,24,25,26]. For a given medium quantity of inflating gas, an initial state A with $\bar{I}=0$ is lower than the red dotted line of its own Maxwell states and is in localized bulging state. With the increase of light intensity, the state moves along the horizontal arrow line due to constant quantity of the inflating gas. With the further increase of the light intensity, the state can move from A to B, which is higher than the blue dotted line of its own Maxwell states and is in the wholly-bulged state. It is concluded that increasing the light intensity can promote bulge propagation, and enables phase transition between homogeneous and inhomogeneous states. In addition, for a given small or large quantity of inflating gas, the balloon without illumination is initially in a homogeneous unbulged state (e.g., state C or E in Figure 13). With the increase of light intensity, the state changes without bulging and phase transition (e.g., from C to D or E to F), in which the deformation of the balloon can be predicted by our homogeneous state-based model.

With the decrease of the light intensity, for a given small or large quantity of inflating gas, the state similarly changes without bulging and phase transition (e.g., from D to C or F to E). However, for given medium quantity of inflating gas, with the decrease of light intensity, an initial wholly-bulged state B can change to locally-bulged state A, or an initial bulged state G can change to another bulged state H. Different from the case of increasing light intensity, the balloon may always be in a bulged state.

**Figure 14:**
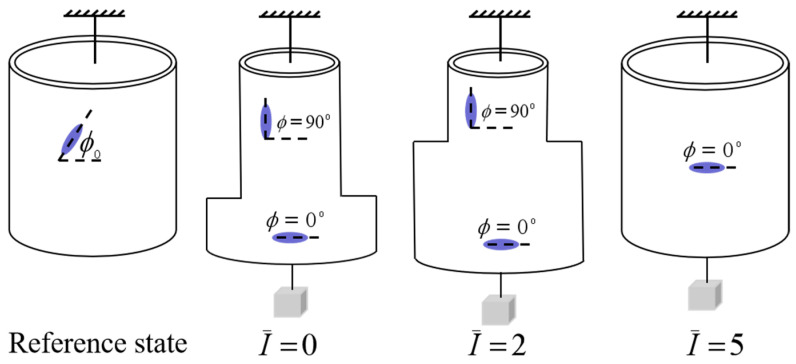
The representative process of a nematic elastomer balloon for initial mesogen angle $\phi_{0}=45^{\circ }$ and axial load $\bar{T}=0.1$ under different light intensities.

Figure 14 presents a typical schematic diagram for the light-activated deformation accompanied with the localized bulging and bulge propagation of the cylindrical nematic elastomer balloon. For an initial localized bulging non-illuminated state, with the increase of light intensity, the bulge propagates and the balloon can be wholly bulged finally, which shows the phase transition between homogeneous and inhomogeneous states in the process of light-activated deformation.

(3) The case of large PV

**Figure 15:**
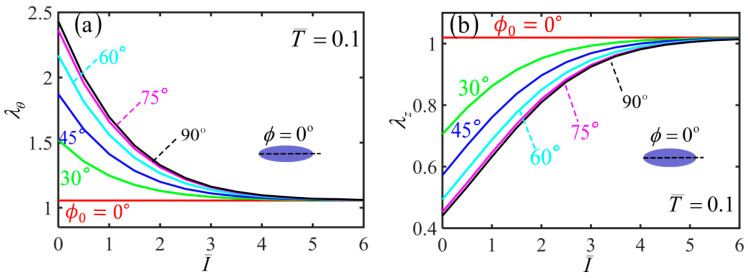
Variations of (**a**) hoop stretch and (**b**) axial stretch with the light intensity of the nematic elastomer balloon with axial load $\bar{T}=0.1$ for different initial mesogen angles.

For a large quantity of inflating gas, the light-activated deformation behaviors of the balloon are similar to the case of the free-standing balloon in Section 3. For $\phi_{0}=0^{\circ }$, both the radius and length of the balloon remain unchanged with the increase of the light intensity. For a nonzero initial mesogen angle, the radius of the non-illuminated balloon has an initial value due to the inflation, which increases with the increase of the initial mesogen angle $\phi_{0}$. For a given nonzero initial mesogen angle, the radius gradually decreases to 1 with the increase of light intensity (Figure 15a). The length of non-illuminated balloon also has an initial value due to the inflation, which decreases with the increase of $\phi_{0}$. For a given initial mesogen angle, the length increases gradually to 1 with the increase of light intensity (Figure 15b).

**Figure 16:**
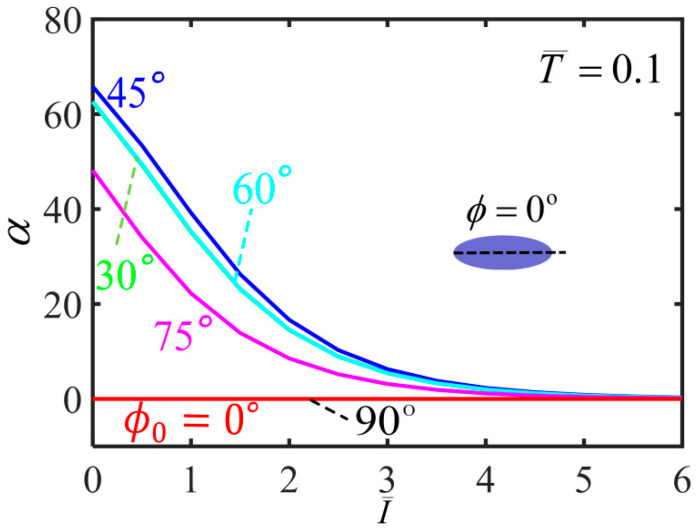
Variations of shearing angle α with the light intensity of the nematic elastomer balloon with axial load $\bar{T}=0.1$ for different initial mesogen angles.

Figure 16 plots the variation of shearing angle with the light intensity during inflation process for different initial mesogen angles. For a given light intensity, the shearing angle firstly increases and then decreases with the increase of the initial mesogen angle, which has the maximum at $\phi_{0}=45^{\circ }$. For $\phi_{0}=0^{\circ }$ and $\phi_{0}=90^{\circ }$, the shearing angle of the balloon is always kept at $0^{\circ }$ with the increase of the light intensity. For $0<\phi_{0}<90^{\circ }$, the shearing angle monotonously decreases to 0 with increasing light intensity. For infinite light intensity, the balloon does not twist because the polymer backbone becomes isotropic.

**Figure 17:**
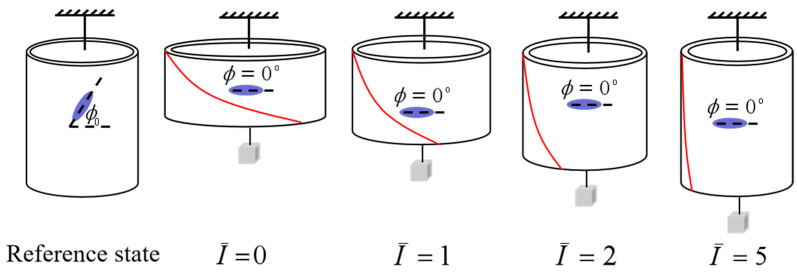
The representative process of a nematic elastomer balloon for initial mesogen angle $\phi_{0}=45^{\circ }$ and axial load $\bar{T}=0.1$ under different light intensities.

Figure 17 illustrates a typical process of a nematic elastomer balloon for initial mesogen angle $\phi_{0}=45^{\circ }$ and axial load $\bar{T}=0.1$. From the reference state to the initial non-illuminated state, the radius of balloon initially increases significantly while the length of the balloon decreases significantly, and the mesogen angle becomes locked at $0^{\circ }$, which is consistent with the reported experiments [15]. With the increase of light intensity, the radius decreases and the length increases asymptotically to 1. The mesogen angle keeps at $0^{\circ }$ and is not affected by the light illumination. The shearing angle monotonously decreases with the increase of light intensity.

## 5. Conclusions

Based on experimental results of light-activated deformation of nematic elastomers, we propose a phenomenological relationship between the light intensity and the material parameter *r* describing the polymer backbone anisotropy. Then, a new theoretical model of optically-responsive nematic elastomer is further established to investigate the light-activated elongation/shortening and twisting behaviors of a cylindrical nematic elastomer balloon. For the free-standing balloon, the light-activated expansion and twisting behaviors are presented and the mechanism is elucidated. For the balloon subjected to a constant axial load, three kinds of light-activated deformation behaviors are investigated. The light intensity and the initial mesogen angle greatly affect the hoop stretch, the axial stretch, the shearing angle and the mesogen angle of the nematic elastomer balloon. In the future, a theoretical model based on an inhomogeneous state needs to be further established to study the bulging of the nematic elastomer balloon. In addition, the deformation behavior of balloon actuators under external torque and torsional restraint deserves to be further explored. It is expected that our results will deepen the understanding of light-activated deformation of the nematic elastomer balloon, and provide guidance for its potential applications in light-activated actuators and machines.

## Figures and Tables

**Table 1:** Material properties and geometric parameters.

Parameter	Definition	Value	Units
T	axial load	0~10	N
P	inflating pressure	0~100	kPa
L	the length of the balloon	100	mm
D	the cross-section diameter	10	mm
H	the thickness	1	mm
$\phi_{0}$	initial mesogen angle	0~90	°
I	light intensity	0~10,000	W/m^2^
r	Light-dependent material parameter	1~5.26	-
μ	shear modulus	0.7	MPa

## Data Availability

The data that support the findings of this study are available upon reasonable request from the authors.
